# Understanding the perspectives of older adults and physiotherapists on home-based lower-limb exoskeletons

**DOI:** 10.1017/wtc.2025.10015

**Published:** 2025-07-14

**Authors:** Reinhard Claeys, Elissa Embrechts, Aikaterini Bourazeri, Ruben Debeuf, Mahyar Firouzi, Matthias Eggermont, Siddhartha Lieten, Benjamin Filtjens, Tom Verstraten, Eva Swinnen, David Beckwée

**Affiliations:** 1 Rehabilitation Research Group, Department of Physiotherapy, Human Physiology and Anatomy, Vrije Universiteit Brussel, Jette, Belgium; 2 Brubotics (Human Robotics Research Center), Vrije Universiteit Brussel, Elsene, Belgium; 3 Center for Neurosciences (C4N), Vrije Universiteit Brussel, Elsene, Belgium; 4 Research Group MOVANT, Department of Rehabilitation Sciences and Physiotherapy, Universiteit Antwerpen, Wilrijk, Belgium; 5 Helmholtz Institute, Department of Experimental Psychology, Universiteit Utrecht, Utrecht, the Netherlands; 6 School of Computer Science and Electronic Engineering, University of Essex, Colchester, UK; 7 Brain, Body and Cognition Research Group, Faculty of Psychology and Educational Sciences, Vrije Universiteit Brussel, Elsene, Belgium; 8 Department of Geriatrics, Universitair Ziekenhuis Brussel (UZ Brussel), Brussels, Belgium; 9 Faculty of Medicine and Pharmacy, Vrije Universiteit Brussel, Brussels, Belgium; 10 e-Media Research Lab, Department of Electrical Engineering (ESAT), KU Leuven, Leuven, Belgium; 11 Department of Engineering Systems and Services, Delft University of Technology, Delft, the Netherlands; 12 Federated Labs AI & Robotics (FLAIR), Elsene, Belgium; 13 Robotics & Multibody Mechanics Research Group, Flanders Make, Elsene, Belgium

**Keywords:** exoskeletons, design, performance augmentation, rehabilitation robotics

## Abstract

Older adults often experience a decline in functional abilities, affecting their independence and mobility at home. Wearable lower-limb exoskeletons (LLEs) have the potential to serve as both assistive devices to support mobility and training tools to enhance physical capabilities. However, active end-user involvement is crucial to ensure LLEs align with users’ needs and preferences. This study employed a co-design methodology to explore home-based LLE requirements from the perspectives of older adults with mobility impairments and physiotherapists. Four older adults with self-reported mobility limitations participated by creating personas to represent different user needs and experiences (i.e., PERCEPT methodology), alongside four experienced physiotherapists who contributed their professional insights. As assistive devices, LLEs were seen as valuable for promoting independence, supporting mobility, and facilitating social participation, with essential activities including shopping, toileting, and outdoor walking. Physiotherapists expressed enthusiasm for integrating LLEs into remote rehabilitation programs, particularly to improve strength, balance, coordination, and walking speed. Key design considerations included a lightweight, discreet device that is easy to don and doff and comfortable for extended wear. Physiotherapists highlighted the potential of digital monitoring to assess physical parameters and personalize therapy. Fatigue emerged as a significant challenge for older adults, reinforcing the need for assistive LLEs to alleviate exhaustion and enhance functional independence. A shortlist of LLE features was drafted and scored, covering activity and design applications. These findings provide valuable insights into the design and usability of home-based LLEs, offering a foundation for developing devices that improve acceptance, usability, and long-term impact on healthy ageing.

## 1. Introduction

Healthy Ageing, as defined by the World Health Organization (WHO), is “the process of developing and maintaining the functional ability that enables well-being in older age.” Within the domain of functional ability, the “ability to be mobile” is identified as a critical subdomain for healthy ageing, essential for maintaining independence, engaging in social activities, and accessing services (WHO, 2015). Data from the 2021 European Green Paper on Ageing emphasizes that 75% of adults over 65 experience at least one difficulty related to physical function, which significantly affects their quality of life (European Comission, 2021).

To promote healthy ageing – particularly functional ability in older adults experiencing declining intrinsic capacities (e.g., decreased muscle strength and/or balance) due to ageing or health conditions (e.g., stroke) – public health interventions are essential (WHO, 2015). These interventions should (1) promote capacity-enhancing behaviors and (2) remove participation barriers by compensating for capacity loss (WHO, 2015). Wearable lower-limb exoskeletons (LLEs) offer a promising solution to support both objectives (Gavrila Laic et al., 2024; Grimmer et al., 2019). Indeed, previous research has shown that LLEs are beneficial for both training and assisting the physical and functional capabilities. Gait training programs using LLEs have been shown to improve gait parameters (Lee et al., 2023), muscle strength (Lee et al., 2023, 2025), endurance (Jayaraman et al., 2022; Lee et al., 2025), energy efficiency (Lee et al., 2023; Martini et al., 2019), functionality (Lee et al., 2023, 2025), and balance (Jayaraman et al., 2022; Lee et al., 2025). As assistive devices, they have demonstrated benefits in increasing self-selected walking speed (Lakmazaheri et al., 2024; Song and Collins, 2021) and reducing the energy cost of walking and stair climbing (Kim et al., 2018; Lakmazaheri et al., 2024; Tricomi et al., 2024). Given their potential to provide both training and assistance, integrated LLE systems are urgently needed to seamlessly combine these functions. However, combining assistive and training goals in a single device presents unique design challenges and trade-offs, such as balancing comfort and wearability for daily use with technical demands like efficient power transmission (Babic et al., 2021). Such systems can enhance mobility and functional independence while ensuring safe and effective use in home environments, supporting ageing in place.

Nevertheless, the field of wearable LLEs is still considered under development, as challenges related to weight, ease of use, adjustability, doffing, and cost persist (Cumplido-Trasmonte et al., 2023). In the context of *assistive* LLEs, developers must determine which activities are most relevant to users to ensure effective support and encourage adoption. Indeed, while LLEs can assist with various activities (Pinto-Fernandez et al., 2020), such as running, walking, and jumping, many of these may not be relevant for older adults. Although studies suggest that LLEs can reduce perceived exhaustion during walking (Galle et al., 2017), it remains unclear how users experience fatigue in daily life and whether exoskeletons should assist with other exhausting activities beyond walking alone.

*Fatigue* is a major barrier to mobility and functional independence, limiting daily activities and reducing overall quality of life (Avlund, 2010; Hardy and Studenski, 2008; Moreh et al., 2010). It encompasses two key constructs: trait fatigue, a chronic state of exhaustion, and fatigability, which is activity-specific (Van Geel et al., 2020). Fatigue affects up to 50% of community-dwelling older adults (Santos-Eggimann et al., 2009), increases fall risk (Blain et al., 2021; Pana et al., 2021), and has been recognized by the WHO as a critical factor in vitality capacity – one of the key physiological determinants of intrinsic capacity, essential for Healthy Ageing (Bautmans et al., 2022).

Furthermore, as *training* devices, LLEs have primarily been used in clinical or laboratory settings with active therapeutic support. For a successful transition to home use and remote rehabilitation, it is essential to explore training methods and principles that facilitate this shift. To aid this transition, integrated LLE sensors are able to monitor various motor performance parameters, enabling both users and healthcare professionals to track physical status in real time (e.g., heart rate, muscle strength, and gait parameters) (Moeller et al., 2023; Netukova et al., 2022). However, as with other design challenges, it is unclear whether target users and their therapists find this feature valuable, and which parameters they would prefer to have assessed and in what manner.

Addressing these challenges efficiently requires the direct involvement of end-users, including both the target group (older adults) and healthcare professionals working with the population, in the development of LLE to ensure alignment with their needs and preferences. While end-user involvement can occur at any stage of development, engaging them in the early design phase – before the device even exists – is crucial for achieving product success and user satisfaction (Kujala, 2003; Shah and Robinson, 2006). To date, few studies have gathered insights from end-users to inform LLE development for older adults. Moreover, existing research involving these groups is largely limited to survey-based studies (Mound and Goher, 2019 and (semi-)structured interviews (Jung and Ludden, 2019; Shore et al., 2022). While these approaches provide valuable insights, they position users in a passive role and fail to adopt a co-design approach, which treats them as active collaborators (Sanders and Stappers, 2008). In co-design, end-users contribute to idea generation, knowledge sharing, and concept development (Sanders and Stappers, 2008), which is essential for improving the usability, acceptance, and overall impact of LLE technology (Carroll et al., 2021).

Therefore, this study aims to use an established active co-design methodology to gather insights from older adults and physiotherapists to inform the development of home-use LLEs. Specifically, the study will focus on an active user-involvement approach that places the needs of the end-user central, making sure that future technology can be developed based on actual user goals, needs, and requirements. Specifically, this study collects insights on:

- the potential use of LLEs as an assistive and training device
- the design and usability requirements for home-based LLEs for assistive and training use
- the added value of assessment of physical parameters while wearing LLEs
- the impact of fatigue on daily life, and the exhausting activities that need support

## 2. Methods

This co-design study is part of the interdisciplinary RevalExo project, aimed at developing a novel LLE to promote healthy ageing for older adults. Ethical approval was granted by the University Hospital Brussels Ethics Committee (BUN: B1432023000271), and the study was registered beforehand on ClinicalTrials.gov (NCT06238206).

### 2.1. Study sample

The study involved two distinct participant groups: older adults and experienced physiotherapists working with the target group. In line with previous co-design studies, a total of four subjects from each group were recruited (Bourazeri and Stumpf, 2018; Neate et al., 2019). This deliberately selected sample of four subjects per group was appropriate given the exploratory nature of the study and the need for in-depth discussions on a complex topic, requiring rich input and active engagement from all participants (Morgan, 1998).

Participants were purposively sampled from the University Hospital Brussels and the network of the research group. All participants received detailed study information and provided written informed consent prior to participation.

Eligibility for older adults to participate required them to be aged 65 years or older, living independently at home, experience some form of mobility challenges during daily living (self-specified), and speak Dutch. Additionally, to ensure that instructions would be understood and participation in group discussion would be possible, participants could show no signs of mild cognitive impairment (Montreal Cognitive Assessment score greater than 23 required) (Carson et al., 2018), and no presence of significant speech disorders. Physiotherapists were eligible if they were actively practicing physical therapy and being experienced treating older adults with mobility impairments, regardless of the patients’ health conditions.

### 2.2. Data collection

This study implemented two distinct co-design approaches to accommodate the specific participant groups. In both approaches, a non-suggestive, open-ended interview guide was used to facilitate discussion (Appendix A – Supplementary materials), with side questions added to maintain focus and minimize irrelevant topics. All sessions were conducted at the Brubotics Rehabilitation Research Center, Brussels, Belgium, and lasted no longer than 2 hr (Krueger, 1998). Discussions were moderated by R.C., with E.E. assisting in note-taking and time management. Sessions were video- and audio-recorded for subsequent thematic analysis.

#### 2.2.1. Older adults

The co-design approach within the older adults group was inspired by the PERCEPT (Persona-Centered Participatory Technology) methodology (Bourazeri and Stumpf, 2018). In this approach, end-users collectively create a set of personas (i.e., hypothetical archetypes representing a broader population – in this case, older adults with some form of functional mobility problems), which are used to examine and discuss user goals and needs (Bourazeri and Stumpf, 2018). The benefits of using personas are well-established, as they enhance focus on user goals, avoid self-referential design, challenge assumptions, provide a common language, and broaden demographic representation (Miaskiewicz and Kozar, 2011; Neate et al., 2019; Nielsen and Storgaard Hansen, 2014).

A total of three separate focus group workshops (W1, W2, and W3) were organized to discuss general, predetermined topics of interest. These topics were shaped by preliminary discussions within the RevalExo consortium, involving both physiotherapists and engineers/developers. In addition, over the three workshops, descriptive data were collected from each participant, including: demographic data (gender, age, use of mobility aid), muscle strength (i.e., Five Times Sit-To-Stand (Csuka and McCarty, 1985)), physical performance (i.e., Short Physical Performance Battery (Guralnik et al., 1994)), and activity levels (i.e., IPAQ-SF (Lee et al., 2011)).

In W1, participants collectively created personas they felt represented the demographic of older adults with mobility problems. Personas’ information included: background information (name, residence, current or most recent job, living situation, goals/motivators, interests, and hobbies), experience with technology, activities of daily living (structured by time of day: morning, noon, afternoon, and evening), and physical challenges. At the start of W2, all participants reflected on the created personas, allowing them to adapt or expand them as necessary. From this point onward, the personas were used as a reference to evaluate the relevance of statements against the broader population and to ensure the generalizability of findings. To continue W2, the impact of fatigue on daily living, and the activities that could benefit from LLE assistance were discussed. To ensure participants understood the concept of LLEs, a brief 3-min introductory video was shown. The video featured five commercially available devices (Keeogo, Hybrid Assistive Limb, EasyWalk, Honda Walking Assist, and Myosuit) across various activities of daily living (ADL) scenarios. It provided limited details to encourage open-ended discussions. Furthermore, as W3 focused on a more in-depth discussion of LLEs, this workshop began with a detailed presentation explaining the different types of systems (assistive, rehabilitation, and augmentation) and introducing a variety of LLEs (eLEGS, Myosuit, Ekso indigo, ReWalk, SMARCOS, Mina v2, ComEx, REX, Hybrid Assistive Limb, BEAR, Ascend, Keeogo, EasyWalk and Honda Walking Assist) through verbal explanations, videos, and pictures. Following this, the design and usability requirements, as well as the assessment methods using LLEs, were discussed. To conclude the final session, participants collaboratively ranked predetermined LLE features, evaluating the importance of exoskeleton characteristics of interest to the RevalExo consortium, including priority activities for assistive LLE support and key design and usability aspects. It was instructed that not all topics could receive the maximum score and a trade-off between features should be made.

#### 2.2.2. Physiotherapists

The co-design approach with the physiotherapists did not incorporate the creation of different personas, given that their experience with the broad patient population would naturally ensure wide applicability. Therefore, the number of sessions for this group was reduced to two (S1 and S2). Hybrid sessions (combined real-life and online) were held to accommodate everyone’s agenda.

S1 began with an in-depth presentation on LLEs, the same presentation used in W3, to ensure that all physiotherapists had a comprehensive understanding of the concept. Following this, discussions focused on the following topics: activities that could benefit from LLE assistance, the design and usability requirements of LLEs, and the key physical parameters for monitoring and evaluation. By the end of S1, the physiotherapists were asked to collectively complete the custom-made survey, rating the importance of predefined exoskeleton features. Similarly to the older adult group, they were instructed to make a trade-off between topics, as not all features could get the maximum score.

In S2, the group discussed the feasibility and practical implementation of home-based training with LLEs. Additionally, they explored the preferred interface for visualizing the key physical parameters identified in S1.

### 2.3. Data analysis

The recordings were transcribed verbatim using Microsoft Word (USA). To ensure consistency, a codebook was established to standardize procedures before transcription (Azevedo et al., 2017). Thematic analysis was performed in accordance with Braun and Clarke’s six-phase approach, which includes familiarizing with the data, generating initial codes, identifying themes, reviewing themes, defining themes, and writing the manuscript (Braun and Clarke, 2006). The analysis was carried out using NVivo 14 (QRS International, USA).

## 3. Results

### 3.1. Older adults (OA)

Four older adults (Table 1) actively participated in the focus group discussions (W1, W2, and W3). Due to unexpected illness, P4 was unable to attend W1 and received an individual booster session to gather the necessary background information and familiarize herself with the concept of personas before joining W2. Similarly, P2 was unable to attend W2, and P3 missed W3, both due to unexpected illness.

**Table 1.** Characteristics of the older adult participants

	P1	P2	P3	P4
Gender	Female	Male	Male	Female
Age (years)	88	83	74	88
MOCA (0–30)	27	25	27	26
5STS (s)	14,64	9,97	23,35	Not able
SPPB (0–12)	8	11	7	5
IPAQ-SF (low - moderate - high level)	Moderate	High	High	High
Walking aid (frequency of use)	None	Walking cane (every day) and walker (every day)	Walking cane (every day) and walker (every day)	Walking cane (every day), ankle orthosis (every day), and wheelchair (only for long distances)

Abbreviations: 5STS, Five Times Sit to Stand; SPPB, Short Physical Performance Battery; MoCA, Montreal Cognitive Assessment; IPAQ-SF, International Physical Activity Questionnaire Short Form.

#### 3.1.1. Personas

The participants co-created a total of three personas: Marcel, Mathilde, and Marie (see Appendix B – Supplementary materials). These fictitious individuals were considered a good representation of the broader demographic, namely older adults with mobility problems, and were used as a reference during the course of the discussions. Each persona included a short biography, some background information, experience of technology usage, activities of daily living, life goals, and physical difficulties.

#### 3.1.2. The potential use of LLEs as an assistive device

LLEs were recognized as tools that increase physical activity and general mobility. Participants noted using them for activities such as going for a walk outside the house, either alone or with others.

“You could say that the exoskeleton stimulates you to move more, that is positive.” (OA P1, W3)

A key benefit of LLEs is their potential to help maintain independence in one’s home environment. Participants highlighted the need for assistance during challenging activities, such as stair climbing, bending the knees to pick up fallen objects, preventing falls, getting up after a fall, and shopping. Shopping involves tasks like covering walking distances, reaching for items on high or low shelves, and handling objects. Concerns about fall prevention were particularly prominent among older adults. Additionally, participants identified the benefits of support for prolonged upright standing and transitioning from a seated position, including transfers from the toilet.

“Prolonged standing. You bump into someone in the store, but you are unable to keep standing. What do you do then? … “Yes, indeed (leave) or search for something to lean against.” (OA P2, W3)

LLEs were also perceived as improving mobility in everyday life by addressing limitations of other mobility aids. For example, they enable easier access to public transportation and escalators compared to walkers.

“With such a mobility aid [referring to LLEs], you get on public transport more easily compared to using a walker. You are no longer so limited.” (OA P3, W2)

#### 3.1.3. The design and usability requirements for home-based LLEs for assistive use

Participants expressed the concern of a bombastic, heavy, and clumsy device, stating the need for a device that is compact and not too heavy. This also relates to their concern about the perception of their use of a mobility aid to get around in their social environment. To counter this, a discrete LLE that can be hidden underneath clothing was proposed.

“Marie (persona 3) doesn’t want to go on the street with her walker because then people see she is not fully mobile. Then she is not going to put *that* on [referring to LLEs as bombastic devices] to go on the streets.” (OA P4, W3)

Donning and doffing should be able to be performed independently, especially relevant for older adults who often are alone, preferably in a seated position to accommodate balance impairments. Additionally, devices that incorporate a backpack module were described as very difficult to put on, due to decreased shoulder mobility, with hip-mounted modules considered a more accessible alternative. The practical aspect of using the device while needing to go to the toilet was also raised as an important consideration.

“Putting on such a backpack (module), I think it is often hard for older adults to put your second arm through.” (OA P1, W3)

Battery requirements were another topic, with participants suggesting that the battery should last for half a day to a full day, depending on the user’s activities. Detachable batteries were deemed feasible, to allow separate charging overnight and to switch out different batteries if needed. Financial concerns were also highlighted, with one participant questioning the affordability and reimbursement of such devices.

“… then you have the next aspect: what is the price of such devices [refers to LLEs]? Who can pay that?” (OA P4, W2)

#### 3.1.4. The added value of assessing physical parameters through LLEs

The use of integrated sensors to monitor physical parameters was generally not of interest to older adults. Participants found such measurement tools difficult to handle and expressed a preference for relying on their own perceptions of physical well-being rather than using technology to quantify it.

“You experience that anyway, whether you progress or decline. That relates to being able to do more or less activities. That sorts itself out.” (OA P4, W3)

It was suggested that while these tools may not appeal to older adults currently, they could become more relevant for future generations with greater technological familiarity. One participant shared a personal example of how a sensor could help alert the wearer to not overexert. Additionally, it was noted that these tools might be of interest for healthcare professionals, such as physiotherapists, to tailor treatments.

“Well, how your situation betters or worsens. That way the physiotherapist can help and tell you what we need to work on.” (OA P2, W3)

#### 3.1.5. The impact of fatigue on daily life, and the exhausting activities that need support

Feelings of fatigue were recognized by the older adults as a significant factor limiting activities of daily living (ADL). To manage these feelings, participants reported adjusting activities by limiting duration (e.g., taking shorter walks with the dog) and spacing them out throughout the day (e.g., scheduling household chores). Regular rest breaks were deemed necessary to prevent and alleviate exhaustion.

“Marie (persona 3), after the morning routines, I think she will start experiencing discomfort in her legs or back due to all the activities” (OA P1, W2). “Yes, the fatigue will start to increase” (OA P4, W2). “So, she will need to learn to pace herself better.” (OA P1, W2)

Participants also noted that energy levels fluctuate throughout the day, with higher energy levels typically in the morning compared to later in the day. As a result, more physically demanding tasks, such as household chores, were intentionally planned for the morning.

“Because she (persona 3: Marie) is alone, doing laundry or cleaning in the morning – how does she do that? I think she would rather do that in the morning because then you have more energy” (OA P1, W2). “Because then you are more fit.” (OA P4, W2)

Fatiguing activities identified by participants included stair climbing and prolonged standing, such as when ironing clothes. Additionally, participants emphasized that the number of repetitions significantly contributes to fatigue, mentioning activities like sit-to-stand movements, crouching, and transitioning from lying to standing.

“When you are lying down and you need to get up, if you do that a couple of times, you are going to feel that. And then you still need to stand up … That is (difficult) due to the exhaustion.” (OA P3, W2)

#### 3.1.6. Ranking of predetermined LLE features by older adults

An overview of the older adults’ rankings of predetermined LLE features is provided in Table 2. Participants placed greater importance on short-distance walking over long-distance walking, as the latter is performed less frequently in their daily lives. The relatively low score of five out of nine for the feature “assist with sit-to-stand” was explained by the availability of environmental support, such as furniture, which many participants use to get up. Furthermore, given that the topic “noise” was ranked last, it is important to note that no reference sound level was provided to the participants, which may have influenced this score.

**Table 2.** Older adults’ and physiotherapists’ rating of LLE features

Topic	Category (A or D)	Older adults score (1–9)	Physiotherapists score (1–9)
Assist with walking short distances	A	9	9
Assist with stair ascent/descent	A	9	9
Comfortable cuffs	D	9	9
Cost	D	9	8–9 (without insurance coverage) or 5 (with insurance coverage)
No obstructed motions while wearing the LLE	D	8	5
Independent use	D	8	9
Easy donning/doffing	D	8	7
Lightweight	D	8	7
Fast donning/doffing	D	7	3
Assistance for 8 hr	D	7	7
Safety	D	7	9
Aesthetics	D	7	8
Assist with walking long distances	A	6	9
Assist with walking down a slope	A	6	7
Able to walk with LLE turned off	D	6	5
Feedback to physiotherapists	D	6	5
Transportability	D	6	6
Assist with sit to stand	A	5	9
Assist with walking up a slope	A	5	7
Assist with crouching/kneeling	A	5	6
Hygiene	D	5	8
Noise	D	4	8
*Additional features?:*			/
*Possible to go to the toilet*	D	9	
*Assist with prolonged standing*	A	8	
*Compactness/ bombastic*	D	8	

*Note:* The category covers whether a topic belongs to the application of activities (A) or design and usability considerations (D).

The feature “assist with crouching/kneeling” sparked discussion among participants. While one participant emphasized its importance for independent living (e.g., picking up objects from the floor), two others deemed it non-essential for LLEs, suggesting alternative solutions like allowing objects to remain on the floor or using tools such as pincher grabbers.

Additionally, the participants identified three user needs that were not part of the original list: “assist with prolonged standing”, “compactness/bombastic design” and “ability to use the toilet while wearing the LLE.”

### 3.2. Physiotherapists (PT)

Four physiotherapists volunteered to take part in this study and participated in both focus group sessions (S1 and S2). All were currently working as a physiotherapist, each with extensive experience treating older adults across different health conditions. Table 3 shows the characteristics of the physiotherapists.

**Table 3.** Characteristics of the physiotherapist participants

	P1	P2	P3	P4
Gender:	Male	Female	Female	Female
Age (years):	34	27	51	36
Practice setting:	Private	University hospital and rehabilitation center	University hospital	General hospital
Main type of patients:	General population	Acquired brain injury	Geriatric population	Acute neurological population
Experience with mobility aids:	Walker and walking cane	Orthosis, exoskeleton, walker, walking cane	Orthosis, walker, walking cane	Orthosis, walker, walking cane

#### 3.2.1. The potential use of LLEs as an assistive device

Physiotherapists emphasized that the primary purpose of LLEs is to enable users to maintain their social and daily activities, thereby mitigating feelings of loneliness and sedentary behavior. While there was some concern that increased assistance might lead to reduced muscle use and faster physical decline over time, it was also acknowledged that LLEs can promote longer-term independence within the home environment and elevate physical activity levels. Key activities identified as benefiting from LLE support include walking, turning, sit-to-stand transfers, picking up objects from the floor, dressing and undressing, stair walking, and using the toilet.

“One of my concerns is that the less we let an older person do, the less they will use their muscles and the faster their muscle loss will be. On the other hand, I understand that it allows someone to live at home longer and be active, so they will use their muscles for longer.” (PT P1, S1)

Regarding walking, physiotherapists highlighted that LLEs should support dorsiflexion to reduce tripping risks, enhance walking speed for tasks such as crossing the street and going to the toilet, and enable to cover longer distances. Especially, the ability to use the toilet while wearing the exoskeleton was deemed essential, given the support needed for standing up, maintaining balance and getting there in time. Improved balance is another crucial feature, with LLEs ideally recognizing and preventing falls, particularly in home settings. For outdoor mobility, navigating uneven surfaces and overcoming obstacles such as front door steps were identified as critical capabilities for the device.

“Independently going to the toilet means that you must be able to go from sitting to standing, so that you must have sufficient strength, that you need to have balance, and a certain walking speed to get there in time.” (PT P3, S1)

#### 3.2.2. The potential use of LLEs as a training device

LLEs were seen as a valuable tool for enabling home-based training without requiring a physiotherapist’s presence. They proposed that the device itself could guide users through integrated instruction features, such as voice commands. However, before allowing independent training, the system should assess health-related factors (e.g., urinary infections) or physical conditions (e.g., poor sleep) that might affect the eligibility for physical activity performance. To achieve this, surveys (e.g., “Do you feel chest pain?”) or wearable sensors (e.g., heart rate monitors) were suggested:

“Maybe you need to do a sort of survey before every training… to see if someone is suitable to do a training.” (PT P1, S2)

Muscle strength training for the lower limbs was identified as a key modality. This could include a mix of isolated movements (e.g., hip flexion-extension, hip abduction-adduction, knee flexion-extension, and dorsi-plantar flexion) and functional exercises (e.g., sit-to-stand, squats, deadlifts, and lunges). Physiotherapists raised concerns about determining the appropriate intensity and duration for strength training, as there is no professional guidance in a home environment. Ideally, the system should adjust resistance to match the user’s capability (i.e., enabling 12 repetitions per set) and progressively increase resistance over time.

“If you go until failure and that is 6 repetitions, then the machine knows I need to lower my resistance. And if that is after 16 repetitions, then it knows I need to increase my resistance a bit.” (PT P3, S2)

Velocity-based training, consisting of executing exercises with a high speed, was stated as a potential solution to these concerns, as execution can stop if a threshold in velocity is reached, while this modality also addresses the parameter power. In addition, blood flow restriction training was also recognized as a promising complementary modality, reducing the need for high loads to obtain a good training effect.

Balance training was another relevant modality for LLEs. The device could act as a safety mechanism, preventing falls while enabling both static (e.g., leaning or reaching in a standing position) and dynamic (e.g., lunges) balance exercises. External feedback could help users correct their posture or shift their base of support.

“Balance exercises with the exoskeleton, where the exoskeleton gives feedback on how the exercise is executed. I think it would be nice if there is some sort of screen that shows with an arrow that you need to move your hip a bit more that way.” (PT P3, S2)

Finally, training to improve coordination and walking speed was suggested. Features such as laser references for shifting endpoints or interval training to alternate walking speeds could enhance these aspects.

“I think it [refers to LLEs] is also ideal to train a bit the walking speed through some sort of interval training with short bouts of walking a bit faster and slower.” (PT P3, S2)

#### 3.2.3. The design and usability requirements for home-based LLEs for assistive and training use

The physiotherapists expressed concern related to the acceptance and willingness to wear the exoskeleton, referring to the experienced barriers with other helping aids such as walkers and orthosis. To address this concern, it was noted that the wearers would prefer it to be discreet, even considering hiding it under clothing, and not being overly conspicuous. However, it was noted that visibility is not entirely negative, as it may encourage others to be more considerate toward the user. If visible, the design should prioritize elegance, sustainable materials, neutral colors, and avoid overly bold features.

“The acceptance we also encounter with orthotics, you have to make sure that it doesn’t get *too* much so that people are still willing to wear it. I think that’s reflected everywhere, that you have to look at the design and that it’s not flashy, but elegant and beautiful.” (PT P4, S1)

Battery performance is critical, with a clear indication of the device’s maximum assistance duration and corresponding walking distance, enabling users to plan social activities confidently. Ideally, the battery should last for a full day, similar to a smartphone you use during the day and charge at night, although options were presented for incorporating a backup battery or wireless charging on the go. The battery pack’s weight should be minimized and carefully balanced to avoid disrupting the user’s center of mass during walking.

“For the user, knowing that they can walk to a destination and back with assistance …” (PT P1, S1) “Yes, because then they can use it during social activities without the fear of ‘What if my battery runs out and I’m still stuck somewhere?’”. (PT P2, S1)

In terms of usability, the exoskeleton must avoid pressure points or tears in clothing. It should be comfortable to wear while seated, lightweight for manipulation and ease of walking, and compact or foldable for transport, such as by car. A pelvis-mounted system is preferred over a backpack design for better practical use. The device should also be easy to don and doff, taking into account stroke survivors with hemiparesis who may need to manage it with one hand. Additionally, it should be weather- and temperature-resistant, hygienic (e.g., washable with interchangeable straps), and sweat-permeable.

“What is important, I think, is that they can sit very comfortably because that is most of what they do after all.” (PT P3, S1)

Lastly, one participant substantiated that the control strategy should allow both the user and the physiotherapist to adjust assistance levels. This enables the user to adapt support based on their condition (e.g., “having a bad day”) and the physiotherapist to make remote adjustments as needed.

#### 3.2.4. The added value of assessing physical parameters through LLEs

The physiotherapists in our study showed great interest in using LLEs to monitor various parameters of their patients. These include: postural sway, walking velocity, muscle strength and power, active range of motions, and weight distribution. The left versus right weight distribution was of interest, especially in conditions such as stroke and hip fractures. It was also proposed that LLEs can estimate physical activity (e.g., “number of steps” and “estimation of activity levels”), including the identification of what activities get performed, and sedentary behavior, not only expressed in minutes but also in sitting bouts. Gait parameters could also inform physiotherapists, evaluating parameters such as stance and swing phase percentages, single and double support time, stride length, and gait symmetry. Fatigue is also a monitorable parameter, recognizing increased effort as users need more assistance after specific activities (e.g. “15 min of walking”) or as the day progresses. In practice, they normally assess this using subjective reports, changes in gait (e.g., shorter steps, foot drop, compensatory movements), and cardiovascular indicators like heart rate (although often less relevant for health conditions with heart medication intake) and respiratory rate. A subtype of fatigue that proved relevant was muscle fatigue, able to be assessed by tracking activities like sit-to-stand transitions or movement velocity.

“How long a person sits. Not only the number of minutes per day, but also the number of sessions. Because sometimes you have people who are active for 2 hours a day, but actually it would be better if they were active for 4 times 30 minutes instead of 1 time 2 hours.” (PT P3, S1)

Feedback from the device to the physiotherapists was seen as beneficial to inform therapy. This included how much support the robot needed to give the user, when and why balance was lost, and why certain activities (e.g., sit-to-stand transfer or walking) were unsuccessful. For example, which muscles contributed the most/least to the movement, and at what point does it go wrong.

“If my patient is doing a sit-to-stand transfer and it goes wrong, it is very difficult for me to see why it goes wrong. What is the quadriceps component here, what is the hamstring component here, what (which muscles) should I train?” (PT P4, S2)

But besides feedback to the physiotherapists, assessment was also seen of interest towards the patients, as a way of setting goals and motivating them. Here, the example of reaching a level of physical activity through the goal of getting a certain number of steps was given.

“I think activity monitoring will be very important because that is going to enhance patient motivation. As my colleagues just mentioned, many patients come in saying, ‘Hey, I did 3,000 steps today’ or something similar. I think that is very important for you as a patient.” (PT P3, S1)

The way the monitored parameters are presented in a user interface is also of great importance. Ideally, the first page of the interface can be tailored to the patient’s specific goals, displaying only the most relevant data while retaining access to comprehensive measurements. Visual tools like graphs showing patient progress over time, with clickable details and reference values (e.g., walking speed norms), allow easy tracking of improvement. These graphs can be adjusted to display daily, weekly, or monthly trends and use color coding to highlight deviations. Data summaries should focus on broader patterns (e.g., preference of gait symmetry over detailed kinematics), making the data more intuitive. Additionally, automated reports summarizing training sessions or weekly activity further support therapy planning and patient monitoring.

“It is interesting to see how the patient’s own values develop, but it would also be interesting to compare the values with normative values, such as walking speed.” (PT P1, S2)

#### 3.2.5. Ranking of predetermined LLE features by physiotherapists

An overview of the physiotherapists’ ranking of predetermined LLE features can be found in Table 2. The topic ‘Cost’ was a point of discussion, designating greater importance when no insurance coverage is provided.

## 4. Discussion

This study gathered valuable insights from two key user groups – older adults with mobility impairments and physiotherapists – to inform the development of home-based LLEs. As co-design is widely recognized as a critical component in assistive technology development (Carroll et al., 2021), the involvement of these user groups is essential. Our findings revealed that while both groups shared common perspectives, they also provided distinct insights on key topics, reinforcing the need for user-centered development. By identifying end-user needs early in the process, developers can ensure that LLEs align with user requirements and mitigate the risk of impractical solutions (Kujala, 2003; Shah and Robinson, 2006).

To structure user input, we applied the PERCEPT methodology with the older adult group, co-creating a set of personas to guide subsequent discussions (Bourazeri and Stumpf, 2018). Contrary to initial concerns that participants would create overly self-referential personas or find the activity trivial, they engaged creatively and embraced the opportunity to explore needs beyond their own. Clear communication about the purpose and relevance of the personas helped anchor discussions in broader user needs and minimized reliance on personal anecdotes. Because participants had co-created the personas, they felt ownership of them and referred to them throughout the sessions, enriching the quality of the dialogues. Nevertheless, as all participants resided in Belgium, the personas they developed likely reflect the specific socio-cultural and infrastructural context of that region, which may limit their applicability to older adults in different geographical or cultural settings.

### 4.1. The potential use of LLEs as an assistive device

Both older adults and physiotherapists recognized LLEs as valuable for promoting independence, supporting mobility, and facilitating social participation. This aligns with prior research highlighting autonomy and social engagement as key motivators for assistive technology adoption (del Rio Carral et al., 2021; Kabacińska et al., 2023).

Our participants identified specific tasks where LLEs could be particularly beneficial, including shopping (walking distances, reaching, handling objects), toileting (balance, sit-to-stand transitions, timely access), and outdoor mobility (uneven surfaces, obstacles, sufficient walking speed). While real-world LLE implementation shows great promise in support of the perspectives from our participants (Basla et al., 2022; Tricomi et al., 2024), future LLEs should be better optimized for daily use, addressing challenging terrains and less common functional activities like lateral stepping, turning, and standing (Pinto-Fernandez et al., 2020). Additionally, despite toileting being crucial for self-independence (Katz, 1983), current LLE designs often overlook this task, making them impractical for bathroom use.

Both study groups highlighted concerns about balance and fall risk, emphasizing the need for LLEs to incorporate balance-related features. Previous research supports this need, identifying fall prevention as one of the most important features of LLEs and noting that users are most likely to wear the device in situations where the risk of falling is highest (Raitor et al., 2024). While evidence suggests LLEs can aid balance recovery (Eveld et al., 2023; Leestma et al., 2024; Monaco et al., 2017), current evaluations are insufficiently addressing this aspect (Pinto-Fernandez et al., 2020). Physiotherapists also raised concerns about muscle disuse, potentially accelerating physical decline (Vallée, 2024; Wall et al., 2014). To mitigate this, LLEs should adopt an “assist-as-needed” approach, providing tailored support that enables movement while encouraging user effort (Naghavi et al., 2020).

### 4.2. The potential use of LLEs as a training device

The LLE training features were assessed with the physiotherapists in our study, given their expertise in rehabilitation. Overall, they supported home-based LLE training to enhance rehabilitation efficiency. A key area of interest was strength training, as LLEs can provide external resistance to human movement. The high relevance of strength training is substantiated by its incorporation in the WHO’s guidelines on Physical Activity and Sedentary Behavior (Bull et al., 2020). Previous studies have demonstrated positive outcomes when integrating resistance in LLE exercises on muscle strength. Lee et al. (2022) conducted a four-week intervention with community-dwelling older adults using a hip exoskeleton providing resistance while walking and found a significant increase in lower limb muscle strength (Lee et al., 2022). Similarly, Fang et al. (2022) implemented a resistance training protocol and reported a 31% increase in plantar flexor strength after 12 sessions of walking with ankle resistance (Fang et al., 2022). While existing research shows great potential, future studies should further explore the use of LLEs for both isolated (e.g., hip adduction-abduction) and functional (e.g., squats, sit-to-stand) strength training exercises.

One major concern raised by participants was how LLEs would determine appropriate training intensities. Strength training is ideally performed until failure, typically reached after 8–12 repetitions per set (Schoenfeld et al., 2021), raising questions about how a device could assess when an individual is sufficiently fatigued to stop. Velocity-based training was suggested as a possible solution and involves executing exercises explosively while using lower resistance levels, making it particularly relevant for home-based training (Schaun et al., 2021). This method also offers an objective way to determine training limits by setting a cut-off at 20% velocity loss (Marques et al., 2020), ensuring that users train at an appropriate intensity.

Beyond strength training, physiotherapists saw potential for LLEs in balance training, particularly when they are able to detect and counter falling when doing the exercises at home. Previous research showed the potential of LLE systems for detecting the occurrence of gait perturbations and for providing assistance to improve stability (Aprigliano et al., 2019; Monaco et al., 2017), but this is yet to be implemented in rehabilitation. Prior research found that gait training with a hip exoskeleton led to improvements in the Berg Balance Scale, a widely used measure of balance performance (Jayaraman et al., 2022; Lee et al., 2022). However, research has largely focused on gait training, and additional investigations are needed to determine whether LLEs could also support balance in other exercise contexts, such as reaching movements. Participants also discussed the potential role of external feedback in enhancing movement execution. While research suggests that external feedback can improve immediate stability, its long-term effects on post-training outcomes remain unclear (Liang et al., 2024).

In addition to strength and balance, physiotherapists highlighted the potential of LLE training for improving coordination and walking speed. Jayaraman et al. (2022) reported that exoskeleton training led to significant improvements in walking speed and the Functional Gait Assessment (Jayaraman et al., 2022). Similarly, Lee et al. (2022) found that older adults who trained with an exoskeleton on stair walking and incline treadmill walking showed significant gains in the Timed-Up and Go test, with those training on an incline treadmill also improving their performance on the Short Physical Performance Battery (Lee et al., 2022).

A key consideration is that current LLE training studies are conducted in controlled settings, such as the hospitals or rehabilitation centers. The physiotherapists in our study raised concerns about how to evaluate whether individuals are capable to engage in active training at home (e.g., chest pain or dizziness). To address this, they advised that home-based training protocols should integrate eligibility assessments, either through self-reported surveys, such as the Physical Activity Readiness Questionnaire (Warburton, 2021), or validated wearable sensors capable of monitoring physical readiness and exertion levels.

### 4.3. The design and usability requirements for home-based LLEs

While the older adult group in our study may be considered active, as indicated by their IPAQ-SF scores, our eligibility criteria and the personas developed were intentionally centered on limitations in functional abilities rather than solely on levels of physical activity. This distinction is important, as functional limitations can be present even in individuals who remain physically active. Our primary interest was in identifying exoskeleton features that address these functional limitations, rather than focusing on features aimed at supporting physical activity per se. Additionally, the persona approach enabled participants to reflect on and consider user needs beyond their own characteristics, including those associated with lower levels of physical activity, ensuring a broader perspective in the prioritization of LLE features.

Older adults and physiotherapists emphasized the need for discrete, compact LLEs to avoid drawing attention to mobility aid use. This aligns with prior research showing that older adults associate mobility aids with ageing and stigma (Jung and Ludden, 2019; Resnik et al., 2009). Future designs should prioritize non-conspicuous aesthetics. Exosuits and soft exoskeletons may be particularly suited to this goal, as they use flexible, textile-based components that can be worn under or integrated into everyday clothing, thereby minimizing visibility and social stigma (Sanchez-Villamanan et al., 2019; Thalman and Artemiadis, 2020).

Easy donning and doffing was found to be critical for independent use, particularly for users with reduced mobility due to ageing (shoulder mobility) or stroke-related impairments (limited hand functioning due to hemiparesis). Participants preferred hip-mounted over back-mounted designs, as the latter often requires assistance from other people or are considered challenging and time-consuming (Basla et al., 2022; Reicherzer et al., 2024). In line with our findings, Jung and Ludden (2019) emphasized that independent donning is essential for home-based exoskeleton technology, necessitating designs that accommodate joint stiffness and limited dexterity (Jung and Ludden, 2019).

Battery requirements included sufficient runtime (half to a full day) and clear visual indicators to provide confidence during activities. Physiotherapists stressed the impact of battery placement on weight distribution and balance, with concerns that back-mounted batteries could shift the center of mass backward. Prior research support these findings, substantiating the importance of clear battery indicators to display remaining support at different walking speeds, and the concern that battery weight could shift the center of mass backward, potentially affecting balance (Vaughan-Graham et al., 2020).

Financial concerns were another key consideration, with older adults questioning their ability to afford LLEs and physiotherapists ranking cost as a top priority if financial support by health insurance is unavailable. Prior research has also underscored the importance of cost concerns, suggesting soft exoskeletons as a more affordable solution due to their lower manufacturing costs (Morris et al., 2023; Thalman and Artemiadis, 2020). While soft exoskeletons may promote adoption and practicality for daily use (easier donning/doffing, don’t constrain degrees of freedom, more lightweight), rigid exoskeletons are capable of transmitting higher torques which may benefit training modalities (Babic et al., 2021). LLEs with a dual-purpose function, for both assistance and resistance, may benefit from a device with combined soft and rigid components. Such systems could incorporate hybrid actuators and user-selectable modes to switch between assistance (e.g., torque support during walking) and resistance (e.g., controlled opposing torques for muscle strengthening), enabling versatile use across various settings, including mobility support and rehabilitation (Fang et al., 2022).

Physiotherapists also highlighted the need for seated comfort, as sitting comprises a significant part of daily life. Back-mounted battery systems, for example, have been found to cause discomfort while sitting during daily and social activities (van Dijsseldonk et al., 2023). However, many current LLEs (e.g., MyoSuit, TWIICE, UGO220) incorporate such a back module. As a result, we suggest reducing the back module size, introducing removable components for seated use, or eliminating the back module altogether.

Finally, adjustable support levels were stated by one of the physiotherapists in this study to accommodate user preferences and expert recommendations. This aligns with user-preferred optimization strategies, where the input of the users steers the assistance control an exoskeleton provides (Ingraham et al., 2023). Previous research in healthy subjects suggests that assistance levels can be reliably identified by the user themselves (Ingraham et al., 2022), but the impact on clinical outcomes is unclear. Future studies should further explore the effects of user preference and the combined role of clinician input as an optimization strategy for LLE assistance (Ingraham et al., 2023).

### 4.4. The added value of assessing physical parameters through LLEs

A key finding from this study was the differing perspectives of older adults and physiotherapists regarding the role of digital monitoring in LLEs. While physiotherapists saw value in tracking physical parameters for motivation and goal-setting in rehabilitation, older adults showed little interest, suggesting that such monitoring is more relevant in rehabilitation than daily life. Previous research highlights the role of external support structures, such as those in rehabilitation, and the perception of added value, to encourage wearable technology use (Moore et al., 2021). Given that our participants, and their personas, were ambulatory and living independently, their intrinsic and extrinsic motivation to use integrated sensors was likely lower. However, wearable activity trackers have been shown to increase physical activity and reduce sedentary behavior (Wu et al., 2023), indicating potential benefits for less-active older adults.

Older adults also viewed integrated measurement tools as more relevant for future generations with greater technological familiarity. This aligns with research showing that perceived technical skills influence adoption (Moore et al., 2021). Designing wearables that match older adults’ abilities and preferences could improve acceptance.

Despite these reservations, both groups recognized the potential of digital monitoring for tailoring treatment and adapting training. Physiotherapists emphasized the importance of tracking walking speed, activity levels, and gait characteristics to provide objective health assessments beyond self-reported data, particularly in real-world settings (Greiwe and Nyenhuis, 2020).

The user interface of monitoring systems was another key concern. Physiotherapists stressed the need for clear, concise data summaries with visual graphs and normative values for easier interpretation. Customization should allow data visualization based on patient goals, while summary reports could aid in tracking progress, adherence, and areas needing adjustment. Prior research confirms that intuitive interfaces enhance task efficiency and reduce cognitive load (Ahmed et al., 2011).

### 4.5. The impact of fatigue on daily life and the potential of assistive LLEs

Older adults in our study acknowledged fatigue as a major barrier in daily life. Feelings of fatigue are known to be highly prevalent, with 27.6–54.5% of community-dwelling older adults reporting exhaustion across 10 European countries (Santos-Eggimann et al., 2009). According to the WHO’s Healthy Ageing framework, fatigue is a key attribute of vitality capacity, which is the physiological determinant of all physical and mental capacities an individual can access at any given time (Bautmans et al., 2022). Participants emphasized exhaustion from daily activities like household chores and stair climbing, aligning with the concept of fatigability – perceived exhaustion linked to activity execution (Eldadah, 2010).

Previous research has shown that LLEs can reduce perceived fatigability during walking (Galle et al., 2017). Extending the use of assistive LLEs to mitigate exhaustion during a broader range of daily activities could potentially enhance functionality and independence in home environments. Therefore, we encourage designers and those evaluating exoskeletons for fatigue reduction to consider support during various daily tasks beyond walking alone, such as stair climbing and cleaning.

Another promising design consideration is the real-time adaptation of exoskeleton support, using fatigue as an optimization objective (Slade et al., 2024). In such systems, the exoskeleton could increase assistance as it detects rising fatigue levels in the user. This aligns with our findings of fluctuating fatigue levels at different times of day (e.g., fitter in the morning) and the exhaustion that follows challenging activities (e.g., stair climbing). Encouraging progress has been made in predicting fatigue using wearable sensors, based on changes in gait parameters and physiological indicators such as heart rate variability, oxygen saturation, and pulse rate (Albert, 2021; Hajifar et al., 2021; Smiley et al., 2023). However, further research is needed to integrate these insights into the control algorithms of LLEs.

## 5. Study strengths and limitations

A key strength of this study is the early-phase involvement of both older adults and physiotherapists, even before a LLE prototype exists. Physiotherapists provided critical insights distinct from those of older adults, including on digital monitoring, user interfaces, and training modalities. While early engagement is essential for effective product development, future work should continuously involve both end-user groups throughout all subsequent stages – design, testing, production, and deployment (Shah and Robinson, 2006). A limitation is the absence of older adults with cognitive impairments, a group that represents a great portion of the ageing population (Pessoa et al., 2019), due to the focus group methodology. While physical demands may be similar, their usability and practical needs could differ significantly. Additionally, the older adults in this study were independently living at home and not undergoing rehabilitation, meaning that perspectives on training needs in a rehabilitative context remained unaddressed. Indeed, based on individual scores on the IPAQ-SF questionnaire, we can state that the older adult group is living an active lifestyle, possibly overlooking the full potential for assistive LLEs to promote mobility. Future research should include less-active older adults, including those participating in rehabilitation programs, to better understand their specific requirements. When involving more dependent older adults in the co-design of assistive technologies, it is also important to consider the perspectives of informal caregivers. These individuals often assist with daily activities, providing valuable insights into the challenges, needs, and priorities of those they care for, and may act as key intermediaries in implementing and managing such technologies. However, previous PERCEPT research has shown that not all care recipients are eager to involve their informal carers in joint discussions during the design process, as they consider themselves to be quite independent (Bourazeri and Stumpf, 2018). Future co-design studies should carefully consider whether caregivers should be integrated into joint discussions with the care recipient – balancing the value of their input against potential social desirability bias – or engaged as a separate target group.

## 6. Conclusion

This study underscores the potential of home-based LLEs to enhance mobility, independence, and social participation in older adults. Through co-design with end-users, we identified key usability requirements: lightweight, discreet, easy to don and doff, and comfortable for extended wear. As assistive devices, they should support essential daily activities like outdoor walking, stair navigation, and toileting. As training tools, they hold promise for improving strength, balance, coordination, and walking speed, though concerns remain about determining appropriate training intensity and user eligibility. While older adults showed limited interest in monitoring physical parameters, physiotherapists valued digital feedback for tailoring therapy. Fatigue was identified as a major barrier, reinforcing the need for assistive LLEs to mitigate exhaustion and support functional independence. By addressing these insights, future LLEs can improve usability, acceptance, and long-term impact on healthy ageing. Future research should incorporate the identified design and activity considerations into the development of home-based LLEs. Upcoming studies should evaluate both assistive and rehabilitation-oriented LLEs in relevant and realistic environments to fully understand their potential.

## Supporting information

Claeys et al. supplementary material Claeys et al. supplementary material

## Data Availability

To protect participant anonymity, the raw and transcribed data from this study will not be shared. The materials, protocols and software code can be made available upon reasonable request to the corresponding author (E.S.).

## Supplementary material

The supplementary material for this article can be found at http://doi.org/10.1017/wtc.2025.10015.
