# Supplementary material for: Understanding the perspectives of older adults and physiotherapists on home-based lower-limb exoskeletons
Source: Wearable Technol. 2025 Jul 14;6:e31. doi: 10.1017/wtc.2025.10015 (PMC12277209; doi:10.1017/wtc.2025.10015)

**Supplementary materials**

Appendix A. Interview guide

| **Older adults** | |
| --- | --- |
| **Workshop 1** | |
| Introduction | *General introduction on the purpose the study and the rules to follow during the group discussions. This is followed by explaining the goal of today's session, and the concept of personas.* |
| **Co-creating the personas: background information** | |
| Transition question | In order to get to know each other we would like you to introduce yourself addressing the following topics: name, residence, current or last profession, living situation, experience with technology, interests and hobbies. |
| Instruction | *Based on the information we just heard from each other, we are going to do the same for our first persona. So think about a typical person from your demographic, an older adult with some form of mobility problem. You can draw inspiration from yourself but also from people from your environment.* |
| Key question | What could be a good name for the persona? |
|  | What is the age of the persona? |
|  | What is the residence of the persona? |
|  | What is his/her current or last profession? |
|  | What is the living situation of the persona? |
|  | Does the persona has experience with technology? |
|  | Does somebody have an idea what the interests and hobbies could be of the persona? |
| **Co-creating the personas: activities of daily living** | |
| Transition question | Before we go further with the personas we would like to know what activities you perform throughout the day. This covers everyday activities both inside and outside the house. We are going to discuss this by collectively going through the day: when you wake up, in the morning, at noon, in the afternoon and in the evening. |
| Key question | Now we want to do the same exercise for the persona. So what activities do you also expect to see with him/her? This could be activities we already heard but this could also be activities that we haven't said yet. |
| **Co-creating the personas: user goals** | |
| Transition question | Next up, we want to know what are your goals in life. Here we mean the things you are striving for. |
| Key question | Now we also want to know the life goals of the personas. Again, don't solely think about your own goals, but try to broaden it. What could goals be for people of your demographic? |
| **Co-creating the personas: physical difficulties** | |
| Transition question | As today's last topic, we are going to talk about physical difficulties and complaints. This can include difficulty in performing activities or tasks, as well as having certain physical complaints. What are difficulties or complains you experience during everyday life? |
| Key question | Now we also want to discuss the physical difficulties and complaints of the personas. This can be things we just listed, but this can also be aspects we didn't hear from you but that we expect to see in a classic older adult with some physical problems. |
| **Co-creating: the same structure in questions is then used to make the following persona(s)** | |
| **Workshop 2** | |
| Introduction | *General introduction on the purpose of today's session.* |
| **Co-creating the personas: reflect on the personas** | |
| Instruction | *In the previous workshop we created these personas (visually represented). First we are going to verbally revise each one of them, afterwards we are going to take time to reflect on them, possible changing or adding information.* |
| Key question | Based on your own experience but also looking at older adults in your environment are there topics that need to be additionally addressed or that need to be adjusted? |
|  | Do you feel like this forms a good representation of the demographic of older adults with mobility problems, or do we wat to create/expand the personas? |
| **Co-creating the personas: the same structure in questions is then used to make the following persona(s) (if stated necessary)** | |
| **Questioning the impact of fatigue** | |
| Instruction | *In the next section, we will take a closer look at the feelings of fatigue. We will use our personas to discuss this. The aim is to define the concept of fatigue from the typical image of an older adult who is functionally limited to some extent. So we try to think not only from our own perspective, but also more broadly.* |
| Transition question | In general, do you think that one or more of the personas experience fatigue? |
| Key question | During which activities do they experience fatigue? |
|  | On the table you see a combination of common daily activities and daily activities we stated during the previous workshop. Do you feel like any of these could cause fatigue? |
| **Questioning the potential of LLEs to support challenging activities** | |
| Introduction | *In order to make sure all participants understand what a LLE is, a brief introductory video was shown, featuring five LLEs in various ADL scenarios. The video provides limited details to encourage open-ended idea generation. Afterwards the personas are again used to open the discussion.* |
| Key question | After the presentation on how LLEs potentially look and work, do you envision activities that would benefit from exoskeleton support? |
|  | Based on the physical difficulties we discussed, could the use of LLEs be of potential help? |
| **Workshop 3** | |
| Introduction | *General introduction on the purpose of today's session. Following with an in depth presentation on the concept of LLEs, with many examples of devices using videos and photos.* |
| **Questioning the design and usability of LLEs** | |
| Introduction | *A moment ago, you were shown a wide variety of exoskeletons. Now the idea is to reflect on the design and usability requirements for the device. We are again using the personas to discuss this.* |
| Key question | First we start with the design, what are the needs and requirements? So related to how the device looks, and how it is drafted? |
|  | Second, we are interested in how convenient it is to use? |
| **Questioning the assessment options using a LLEs** | |
| Introduction | *Within the exoskeleton there is the possibility that we can measure different physical parameters. Think about the possibilities that exist using a smartwatch of smartphone but with more options.* |
| Key question | Are there parameters that are relevant/interesting to assess? |
|  | With the parameters we addressed, how would it be best to visualize them in an interface? |
| **Questionnaire scoring the importance of LLE features** | |
| Introduction | *Beforehand, based on what the researchers expected to be of interest, we made a survey with different topics related to exoskeleton features. We are going to score each topic collectively again taking the perspective of the personas into account.* |
| Key question | How important (score from 1 through 9) is the following topic? |
| **Physiotherapists** | |
| **Session 1** | |
| Introduction | *General introduction on the purpose the study and the rules to follow during the group discussions. This is followed by explaining the goal of today's session, including an in depth presentation on the concept of LLEs, with many examples of devices using videos and photos.* |
| Opening question | In order to get to know each other, I would like to ask that everybody introduces their selves in a couple of sentences. |
| **Questioning the potential of LLEs to support daily activities** | |
| Instruction | To start off we would like to discuss the activities of daily living, where LLEs may potentially help the wearer to increase functionality. |
| Key question | What are the activities of daily living that need additional support? To enhance the functionality and independence of a person? The activities that enable individuals to stay longer in their home environment (age in place)? |
| **Questioning the assessment options using a LLEs** | |
| Introduction | A lower-limb exoskeleton can be used in the home environment, able to assist the wearer in daily activities and as a remote rehabilitation tool. Here, it can also evaluate the physical state of the wearer through integrated sensors. |
| Key question | What information is relevant for a physiotherapist to monitor? During what moments in time? |
| **Questioning the design and usability of LLEs** | |
| Introduction | *At the start of today's session, you were shown a wide variety of exoskeletons. Now the idea is to reflect on the design and usability requirements for the device.* |
| Key question | What are the requirements for exoskeleton design before it is actually usable? Topics such as aesthetics, donning/doffing, weight, transportability, battery life, etc. |
| **Questionnaire scoring the importance of LLE features** | |
| Introduction | *Beforehand, based on what the researchers expected to be of interest, we made a survey with different topics related to exoskeleton features. We are going to score each topic collectively .* |
| Key question | How important (score from 1 through 9) is the following topic? |
| **Session 2** | |
| Introduction | *General introduction on the purpose of today's session.* |
| **Questioning training with a LLE** | |
| Instruction | *LLEs have the ability to allow remote rehabilitation. We are going to discuss your opinion and insights in this modality.* |
| Key question | Is this a useful modality? What is your conception of the idea? |
|  | Which exercises do you want to see incorporated? |
| **Questioning the interface (as a follow-up on the assessment)** | |
| Instruction | *In the previous session you stated a lot of assessment needs to be integrated in a LLE. We have bundled this information, and we will present it to you. Now we want to know what the user interface should be?* |
| Key question | How should the assessed parameters best be presented to you? How is this best done? Also related to visualization and time? |


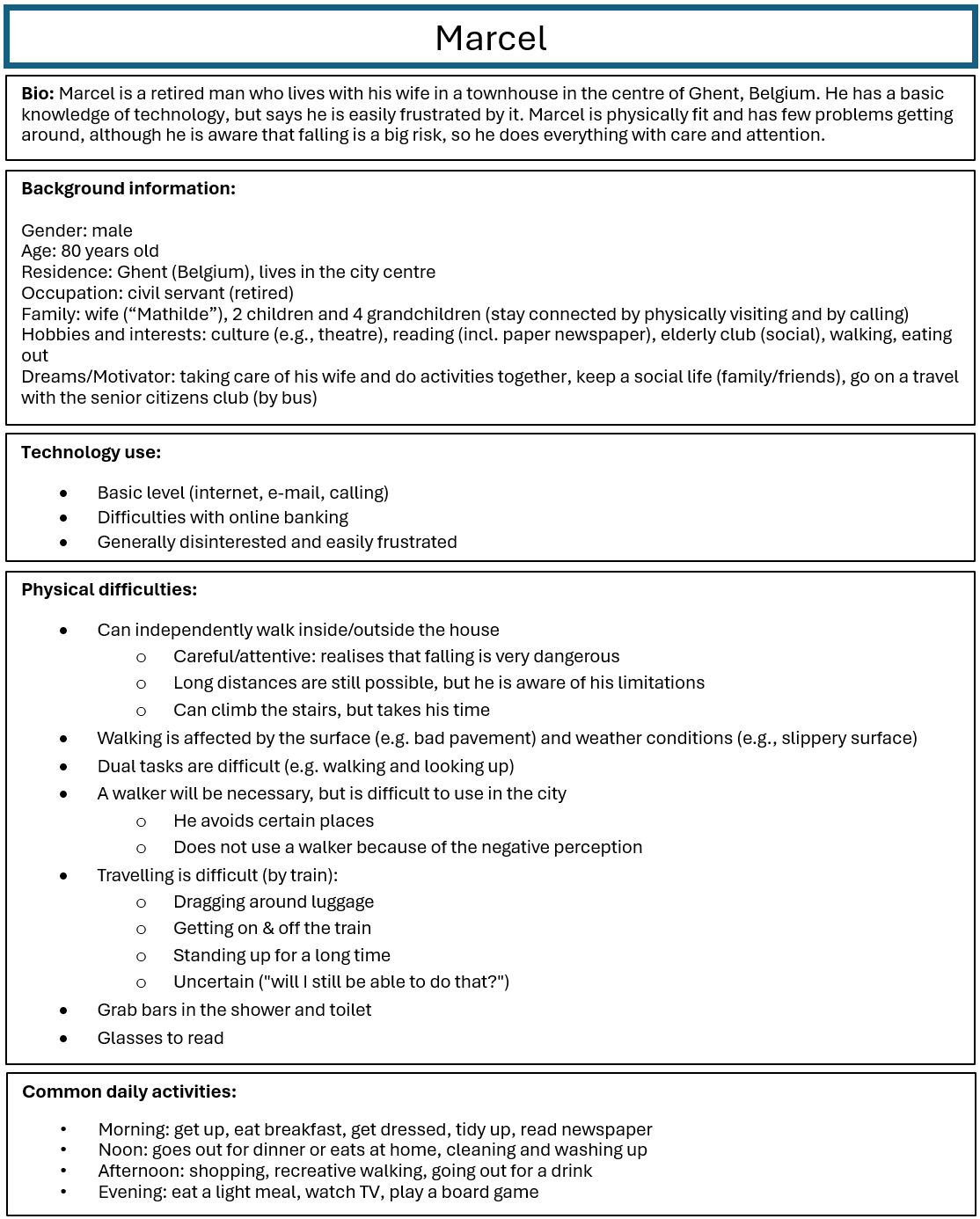
Appendix B. Personas


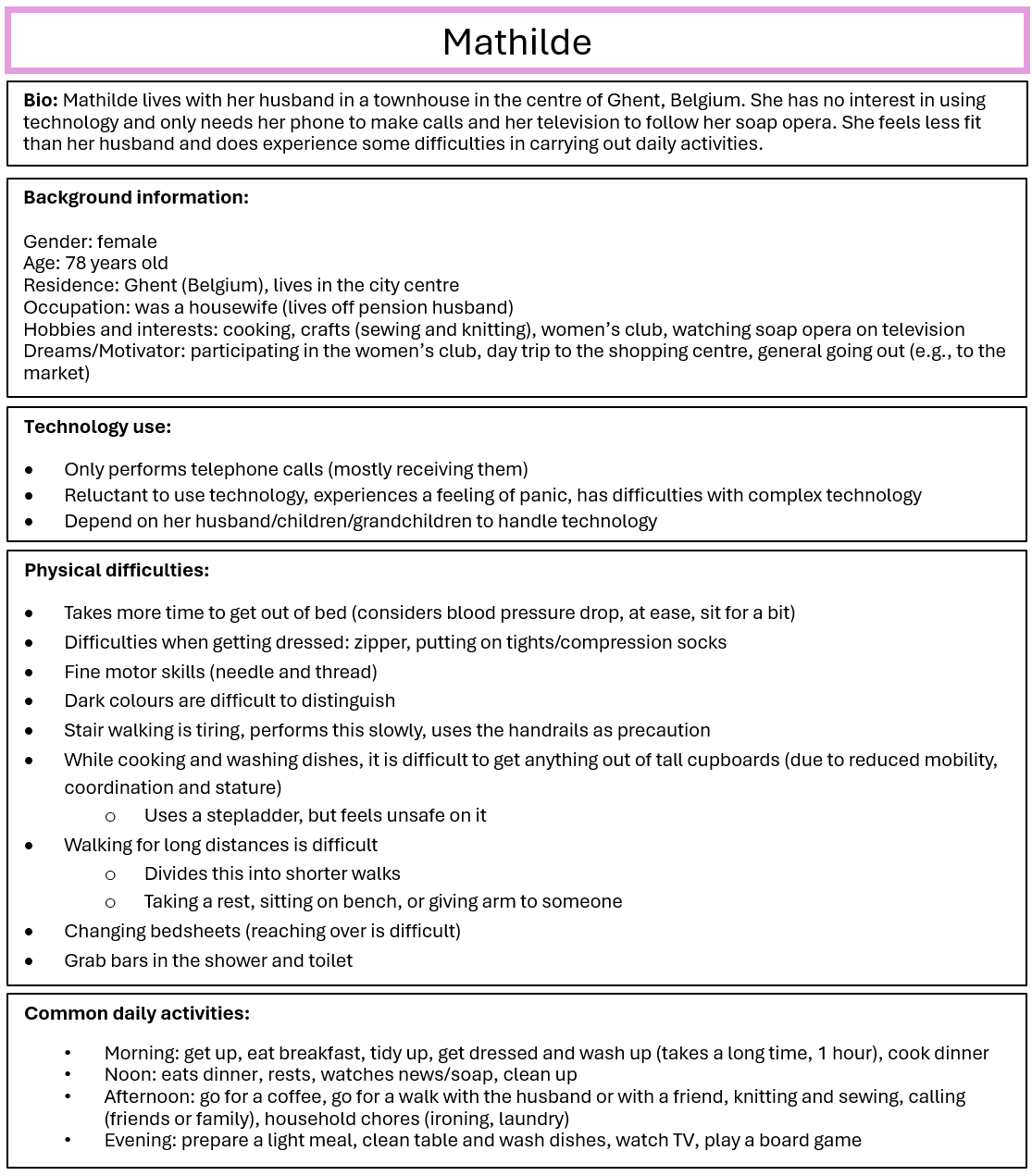


**Common daily activities:**

- Morning: get up, eat breakfast, get dressed, tidy up, read newspaper
- Noon: goes out for dinner or eats at home, cleaning and washing up
- Afternoon: shopping, recreative walking, going out for a drink
- Evening: eat a light meal, watch TV, play a board game

**Common daily activities:**

- Morning: get up, eat breakfast, get dressed, tidy up, read newspaper
- Noon: goes out for dinner or eats at home, cleaning and washing up
- Afternoon: shopping, recreative walking, going out for a drink
- Evening: eat a light meal, watch TV, play a board game


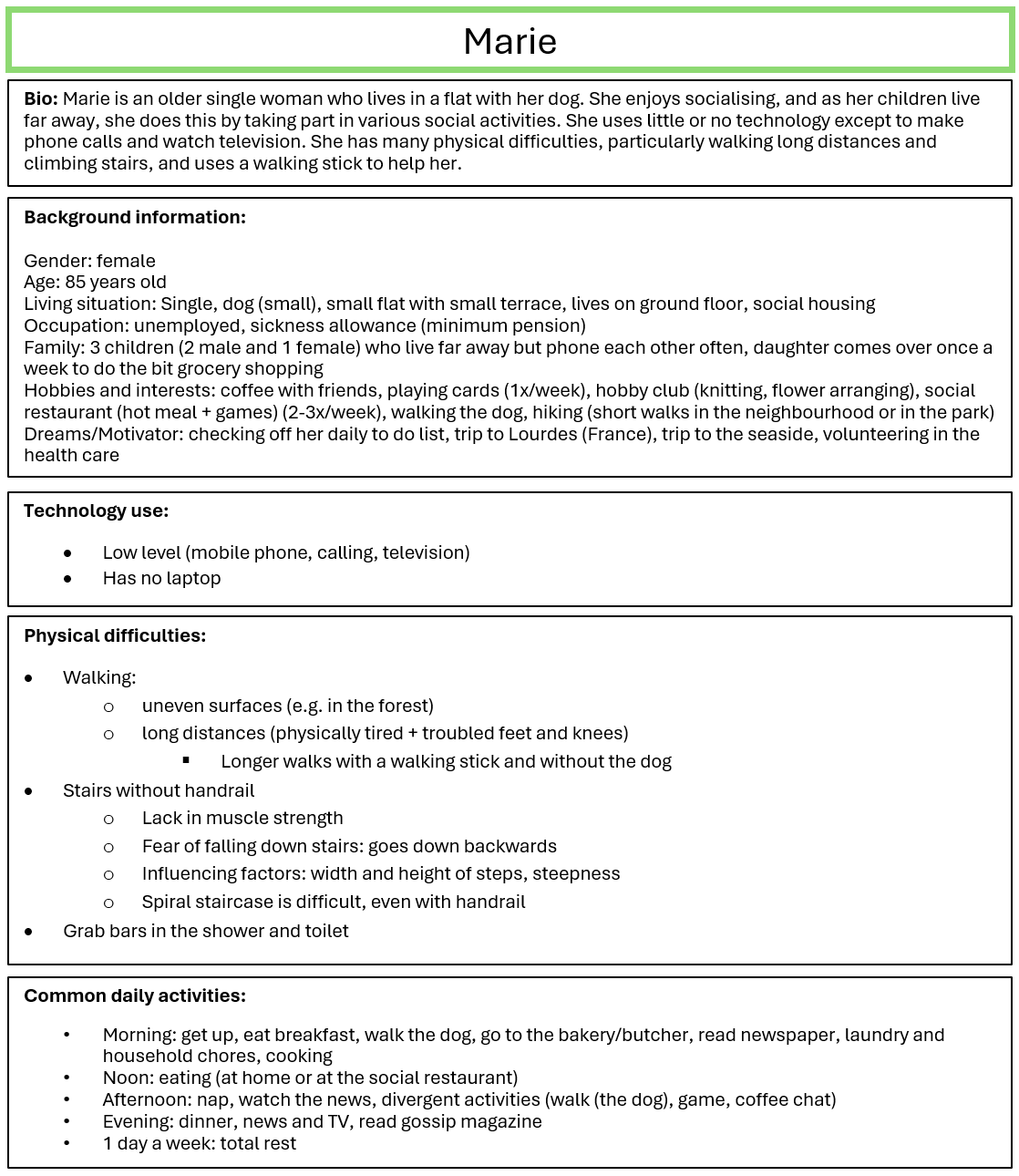

Supplement: Claeys et al. supplementary material [file S2631717625100157sup001.docx]
